# Leukocyte Telomere Length as a Molecular Biomarker of Coronary Heart Disease

**DOI:** 10.3390/genes13071234

**Published:** 2022-07-12

**Authors:** Olga V. Zimnitskaya, Marina M. Petrova, Natalia V. Lareva, Marina S. Cherniaeva, Mustafa Al-Zamil, Anastasia E. Ivanova, Natalia A. Shnayder

**Affiliations:** 1 Department of Outpatient Therapy and General Practice with Course of Postgraduate Education, V.F. Voino-Yasenetsky Krasnoyarsk State Medical University, 660022 Krasnoyarsk, Russia; stk99@yandex.ru; 2 Department of Therapy, Faculty of Postgraduate Education, Chita State Medical Academy, 672000 Chita, Russia; larevanv@mail.ru; 3 Department of Internal and Preventive Medicine, Central State Medical Academy of the Presidential Administration, 121359 Moscow, Russia; doctor@cherniaeva.ru; 4 Department of Physiotherapy, Faculty of Continuing Medical Education, Peoples’ Friendship University of Russia, 117198 Moscow, Russia; alzamil@mail.ru; 5 V.M. Bekhterev National Medical Research Center for Psychiatry and Neurology, Institute of Personalized Psychiatry and Neurology, 192019 St. Petersburg, Russia; anastasiae.ivanova@bekhterev.ru

**Keywords:** telomere length, stable coronary heart disease, people, adults, coronary atherosclerosis, acute coronary syndrome, acute myocardial infarction, early vascular aging, molecular predictors

## Abstract

Background. This work is a review of preclinical and clinical studies of the role of telomeres and telomerase in the development and progression of coronary heart disease (CHD). Materials and methods. A search for full-text publications (articles, reviews, meta-analyses, Cochrane reviews, and clinical cases) in English and Russian was carried out in the databases PubMed, Oxford University Press, Scopus, Web of Science, Springer, and E-library electronic library using keywords and their combinations. The search depth is 11 years (2010–2021). Results. The review suggests that the relative leukocyte telomere length (LTL) is associated with the development of socially significant and widespread cardiovascular diseases such as CHD and essential hypertension. At the same time, the interests of researchers are mainly focused on the study of the relative LTL in CHD. Conclusions. Despite the scientific and clinical significance of the analyzed studies of the relative length of human LTL as a biological marker of cardiovascular diseases, their implementation in real clinical practice is difficult due to differences in the design and methodology of the analyzed studies, as well as differences in the samples by gender, age, race, and ethnicity. The authors believe that clinical studies of the role of the relative length of leukocyte telomeres in adult patients with coronary heart disease are the most promising and require large multicenter studies with a unified design and methodology.

## 1. Introduction

Cardiovascular diseases (CVDs) are prevalent worldwide. In Europe, there were 19.9 million new cases of cardiovascular disease in 2017. There were 2.5 million new cases of CVD in Germany, 1.115 million in France, and 1.209 million new cases of CVD in the United Kingdom [1]. CVDs are the leading cause of death in most European countries [1] and Russia [2]. In European countries, CVDs cause about 2.2 million female and 1.9 million male deaths per year [1], and in Russia, the mortality rate from CVDs is about 938,500 per year [3]. Coronary artery disease (CAD) or coronary heart disease (CHD) are responsible for over 50% of CVD deaths in Russia [2,3] and cause about 40% of CVD deaths in Europe [1].

Genetic factors have a significant impact on the risk of CVD. A history of CVD increases their future risk from 40% to 75% depending on the degree of the relationship [4]. Aging is a major risk factor for CVDs and cerebrovascular diseases but it has been established that people age at different rates. Therefore, aging is characterized by chronological and biological aging. Chronological aging refers to the time elapsed since a person was born, and biological aging refers to the decline in the function of a person’s tissues and organs. In people who age normally, chronological age is equated with biological age [5]. In 2008, Nisson et al. [6] formulated the concept of early vascular aging (EVA), according to which the biological age of a person depends on the age of his or her blood vessels, and persons with EVA syndrome due to early vascular aging have an increased risk of developing CVDs and their complications. There is no clear list of EVA criteria, but some authors refer to physiological biomarkers of EVA as increased arterial vascular wall stiffness (arterial stiffness) assessed by pulse wave velocity or by calculation of the cardiovascular ankle index; a thickening of the intima-media complex; endothelial dysfunction [7,8,9]; the presence of atherosclerotic plaques in arteries; and the deposition of calcium phosphate crystals in arterial intima [10,11].

Deoxyribonucleic acid (DNA) methylation and telomere shortening are considered potential molecular biomarkers of EVA [5]. In a progressively aging world population, early diagnosis based on the development and implementation into real clinical practice of such molecular biomarkers is very important, as it may allow effective identification of people with a high risk of EVA in different climatic–geographical regions and racial and ethnic groups. Such a personalized strategy is expected to reduce the socio-economic burden of age-associated diseases including CVDs in general but in particular CAD.

In recent years, blood leukocyte telomere length (LTL) has been considered a “mitotic clock” fixing human biological age [12] and as a potential molecular biomarker of EVA, but research in this area is ongoing and no unequivocal decision on this issue has been made yet [13].

Also, as other early prognostic biochemical biomarkers of EVA, CVDs (atherosclerosis and CAD) are being actively studied: neutrophil gelatinase-associated lipocalin (NGAL) [14]; tissue inhibitor of metalloproteinase 2 (TIMP-2) [14]; fibroblast growth factor 23 (FGF-23) [15,16]; syndecan-1 [16]; interleukin 6 (IL-6) [17]; and galectin-3 [18].

In addition, biomarkers of the adverse outcomes of atherosclerosis and CAD (total mortality and mortality from CVDs) are being developed, among which LTL is of undoubted scientific and clinical interest [19,20]. This is due to the fact that the mechanisms of LTL shortening in adults, which lead to stable CAD, acute coronary syndrome (ACS), and acute myocardial infarction (AMI), have not yet been sufficiently studied [21]. LTL is also actively studied in other CVDs (essential arterial hypertension [22,23], atrial fibrillation [24,25,26,27], cardiomyopathy [28], cerebrovascular diseases (stroke [29,30,31,32,33], vascular cognitive disorders [34,35], and vascular dementia [36]).

**Figure 1:**
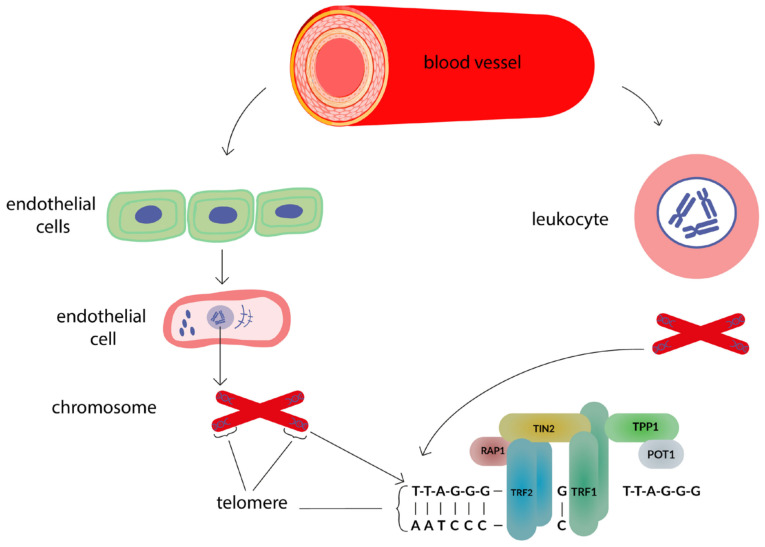
The relationship between the telomere length of endotheliocytes and leukocytes.

Telomeres (from the Greek telos “end” and meros “part”) are nucleoprotein structures located at the ends of chromosomes (Figure 1), consisting of a noncoding repetitive DNA sequence (-TTAGGG-), a single-stranded region called the protruding part of the G-chain [37,38], and proteins that compose the Shelterin complex. Due to the Shelterin proteins, telomeric DNA is folded into a complex three-dimensional structure [39,40]. The telomere length of an adult human is approximately 10–15 thousand base pairs (bp). The protruding part of the G-chain, including 150–200 bp, can bend and form a loop structure (T-loop). Also, a D-loop can be formed [39]. The T-loop protects the 3’OH ends of the chromosomes from recognition of 3’OH as a double break in the DNA chain [40,41].

Telomeres are bound to telomere-specific proteins that are part of the Shelterin complex. The Shelterin complex provides greater telomere stability and consists of six telomere-specific proteins: Telomeric Repeat Binding Factor 1 (TRF1); Telomeric Repeat Binding Factor 2 (TRF2); Telomeric Interacting Nuclear Factor 2 (TINF2 or TIN2), a complex consisting of TERF1 and Nuclear Factor 2; Protection of Telomeres 1 (POT1), a protein that provides telomere protection; Shelterin complex subunit and telomerase recruitment factor (TPP1), a subunit of Shelterin complex and telomerase recruitment factor; and TERF2 interacting protein (TERF2IP or RAP1), a protein that interacts with TERF2. All six proteins regulate telomere length [42].

The interaction of the Shelterin protein complex with the telomere DNA sequence ensures the stabilization of the telomere structure and regulates the access of proteins involved in DNA elongation and repair. The TRF1 complex is involved in telomere length control by regulating telomerase access to the telomere sequence [42]. The TRF2 complex protects the G-chain protrusion from degradation and prevents telomere fusion [43].

Telomeres were first identified by Hermann Müller in 1938 and he and McClintock determined the protective role of telomeres in 1941 [44]. The first human telomeres were isolated by Moyzic et al. [45] in 1988. Since then, telomere biology has been extensively studied.

Telomeres protect chromosome ends, maintaining genome integrity and stability [38]; telomeres prevent loss of coding DNA during DNA replication [39]. Chromosome telomere fusion can lead to gene amplification, chromosome imbalance, non-reciprocal translocations, and changes in gene expression [40,45]. Telomere length is shortened due to end replication problems and nucleolytic DNA degradation. Each cell division results in the loss of 50–200 bp of telomere sequence [40,46,47]. It is believed that telomere shortening is the reason for the limited number of divisions in most human cells. This phenomenon was first described by Hayflick [48] on diploid human cells. Also, he found that each cloned cell in the population is endowed with the same doubling potential—50 ± 10 cell divisions. This phenomenon was later called the “Hayflick Limit,” meaning that a cell can divide a limited number of times after which cell division stops [49].

In contrast to most somatic cells, hematopoietic stem cells, germ cells, keratinocytes in the basal layer of the epidermis, uterine endometrial cells, and cells from various tumors avoid telomere shortening by activating telomerase [40]. Telomerase, also called terminal transferase, is a ribonucleoprotein that adds a species-dependent telomere repeat sequence to the 3’ end of telomeres [50]. Although telomerase activity has been vigorously investigated over the last few decades, many questions remain open regarding the mechanisms of physiological regulation in normal cells [51]. The complex regulation at the levels of transcription, splicing, and post-transcriptional activation certainly contributes to that. Moreover, mutational analysis and knockdown experiments showed that telomerase deficiency led to telomere loss and uncapping, causing progressive atrophy of renewal tissues, a gradual depletion of stem cells, and the eventual failure of organ systems. Above all, telomerase may play a critical role in cellular and organismal aging and could be a potential target for anti-aging therapies [52].

Telomere shortening is associated with non-genetic (physiological) and genetic mechanisms of aging (inflammation, oxidative stress, chronic diseases, cellular aging, mortality), as well as with social factors of aging (gender, race, ethnicity, low socioeconomic status, stress, smoking) [47,53]. EVA syndrome, as well as age-associated diseases (CVDs, type 2 diabetes mellitus, cancer, or chronic obstructive pulmonary disease), are associated with telomere shortening and/or dysfunction [46]. For example, people with different degrees of atherosclerosis and CAD have significantly different LTL [52].

The purpose of this systematic review is to find, analyze, and systematize studies on the relationship between LTL and CAD.

## 2. Materials and Methods

Full-text publications were searched in the following databases: PubMed, Web of Science, Springer, Google Scholar, Oxford Press, Clinical Cases, Cochrane, and e-Library. We analyzed articles published between 10 January 2010 and 10 December 2021. Key words and their combinations were used to search for: “telomere length,” “stable coronary heart disease,” “humans,” “adults,” “coronary atherosclerosis,” “acute coronary syndrome,” “acute myocardial infarction,” “early vascular aging,” and “molecular predictors.”

**Figure 2:**
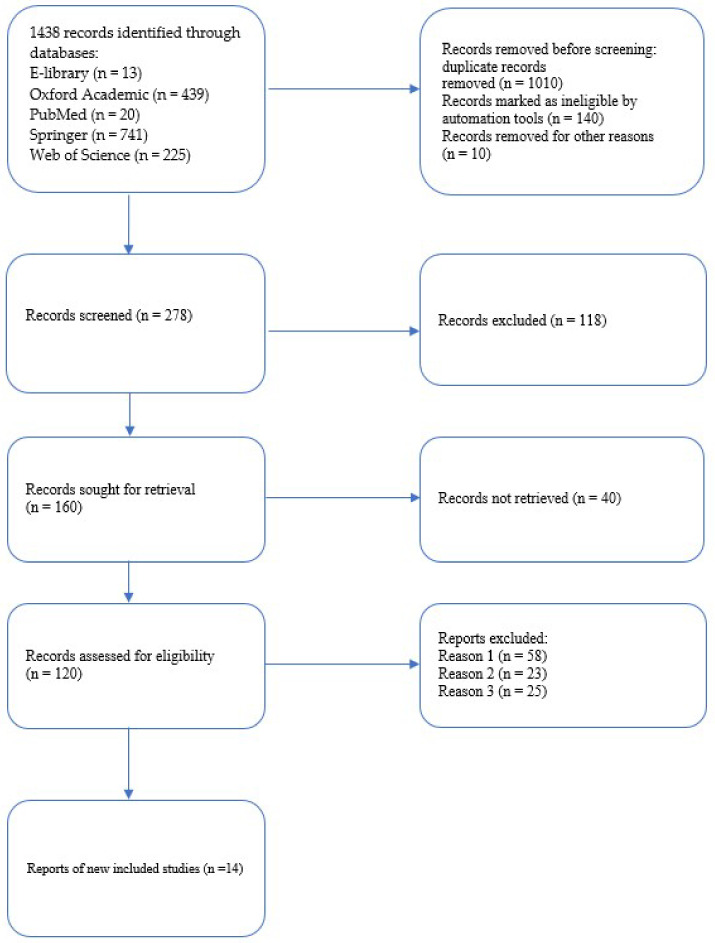
Flow chart diagram visualizing the database searches and the number of publications identified, screened, and the final full texts included in the present systematic review. Reason 1—there is no primary data on relative LTL in the article. Reason 2—the method of measuring relative LTL was carried out using an alternative method to the Ca. thon R. M. method. Reason 3—review and meta-analysis.

Publications were searched and selected using the Preferred Reporting Items for Systematic Reviews and Meta-Analyses (PRISMA) 2020 guidelines. A total of 278 articles were found using the keywords (Figure 2). We analyzed original studies, systematic reviews and meta-analyses, clinical cases, and Cochrane reviews. After reading the relevant titles and abstracts, we excluded publications with irrelevant topics as well as duplicate publications, open access preprints, and conference posters. We excluded original studies that did not provide primary data, including demographic data, and original articles with retrospective studies. We also excluded reviews, editorials, animal experiments, and publications with questionable or insufficiently proven results of the authors’ investigated molecular biomarkers of CAD and the relationship between CAD and LTL. In addition, we analyzed earlier publications of historical interest.

Finally, we selected 14 suitable publications for our systematic review in which the authors used comparable methodological approaches to measure LTL using a T/S ratio and/or bp. This approach was important to be able to systematize the results of the analyzed studies. We analyzed but excluded from further processing publications with alternative methods of LTL calculation.

We selected articles in which the relative LTL was measured by the method of Cawthon R. M. [54]. This technique uses real-time quantitative polymerase chain reaction (PCR) to measure LTL. The advantages of this technique are speed of performance and a small amount of DNA. Relative LTL is defined as the ratio of telomeric repeats to a single copy of a standard gene (T/S) and is measured in conventional units (CU). The T/S ratio reflects the average LTL in all human leukocytes [55].

Statistical analysis of the obtained data was performed using the SPSS software package, version 23 (Stat Soft, Tulsa, AK, USA,). Since the size of compared samples was small (n ≤ 30), nonparametric statistics were used. Median (Me) and percentiles [25; 75] were calculated for each of the three groups (healthy adults without CVDs, adults with stable CAD, and adults with AMI). The groups were compared using the Mann–Whitney test. Significance of differences was considered significant at a *p*-value < 0.05.

## 3. Results

### 3.1. Leukocyte Telomere Length in Patients with Stable Coronary Heart Disease

Our analysis of the studies demonstrates that the issue of LTL changing in patients with CAD has been extensively studied worldwide. The number of LTL studies on this disease is increasing in different age groups of patients and in different racial and ethnic groups compared with healthy controls (Table 1). Thus, in healthy adults without CVD, the relative LTL ranged from 0.69 [56] to 1.52 CU [55] and the median relative LTL was 0.93 [0.70; 1.10] CU.

Hassler et al. [56] studied relative LTL in healthy adults (mean age of men 40.77 ± 11.62 years, mean age of women 44.71 ± 10.96 years). The authors found no significant difference in relative LTL in men and women of the study age (0.70 ± 0.28 vs. 0.69 ± 0.31 CU, *p*-value = 0.75).

In patients with stable CAD (Table 2), the relative LTL ranged from 0.82 [63] to 1.13 CU [55]; the median relative LTL was 0.86 [0.82; 1.07] CU. The differences in median LTL in patients with stable CAD compared to healthy controls were not statistically significant (*p*-value = 0.850).

Mazidi et al. [62] found that older men without CAD had a shorter relative LTL than younger men without CAD (6380 ± 80 bp vs. 12,420 ± 60 bp, *p*-value < 0.05).

Willeit et al. [55] in a prospective population-based PCR study, evaluated relative LTL in individuals aged 45 to 84 years without CVD and in patients with stable CAD. The authors found that LTL in patients with stable CAD was significantly shorter than in healthy individuals of the same age (1.13 ± 0.52 CU in patients with CAD versus 1.52 ± 0.81 CU in healthy individuals, *p*-value < 0.001). An interesting finding was that LTL was shorter in men than in women (1.41 [1.33–1.49] CU versus 1.55 [1.47–1.62] CU; *p*-value = 0.02). The authors attributed this phenomenon to the higher estrogen levels in women. In addition, the study found that LTL was inversely correlated with age (r = −0.22, *p*-value < 0.001).

The possibility of using LTL as a molecular biomarker of human biological aging as it reflects the telomere length of endothelial cells has been confirmed by several studies. The study of Hammadah et al. [63] established the relationship between LTL shortening and low level of CD34+ expression on human endothelial progenitor cells. After adjustment for age, sex, race, body mass index, smoking, and previous myocardial infarction, a shorter LTL was associated with lower CD34+ cell levels; for every 10% shorter relative LTL, CD34+ levels were 5.2% lower (*p*-value < 0.001). This is indirect evidence of the decreased regenerative capacity of bone marrow cells and the decreased repair of blood vessel endothelium. A study by Wilson et al. [66] revealed a strong correlation between endotheliocyte telomere length and relative LTL in the blood (r = 0.62, *p*-value < 0.001). Thus, LTL reflects vascular endotheliocyte telomere length, which allows us to use LTL assessment as a biomarker of vascular age, EVA, and human biological aging in various CVDs in adults.

Starnino et al. [61] found that patients with stable CAD had a shorter LTL compared with healthy volunteers and people without CVD (*p*-value < 0.001).

Tian et al. [58] analyzed cases of stable CAD in men younger than 55 years and women younger than 65 years. The authors measured relative LTL in Chinese patients with premature CAD compared with a control group (people of comparable age without CAD). The effects of oxidative stress on LTL shortening were assessed. Patients with premature CAD had a shorter relative LTL compared with those without CAD (0.88 ± 0.86 CU versus 1.1 ± 0.57 CU, *p*-value = 0.015). Thus, there was an association between LTL shortening and decreased plasma antioxidant capacity in patients with CAD.

Huang et al. [66] studied the relationship between LTL and all-cause mortality, cardiovascular mortality, and cerebrovascular mortality among adults in the USA. The study included 7827 participants (48.18% men). The researchers conventionally divided all participants by LTL into three groups: short LTL (0.77 ± 0.09 CU); medium relative LTL (1.00 ± 0.06 CU); and large LTL (1.32 ± 0.26 CU). After 158.26 months of follow-up, there were an average of 1876 (23.97%), 87 (1.11%), and 243 (3.10%) all-cause, cerebrovascular, and cardiovascular deaths. The authors showed that LTL was nonlinearly correlated with all-cause mortality (OR—95% CI: 0.03 to 0.09; *p*-value < 0.0001) but not with mortality from cerebrovascular disease and CVDs (*p*-value *> 0.05*).

Yakhontov et al. [64] studied the relationship between LTL in men with stable angina I-III functional classes according to the Canadian Cardiovascular Society classifications [67] in different age groups: the middle-aged group (median age 52 [46.5; 55] years) and the elderly group (median age 64 [62; 67] years). The authors found no statistically significant differences in LTL in patients with stable angina pectoris as a function of mean and old age (*p*-value = 0.058) [63]. Another study by these authors [64] examined LTL in young (median age 52 [46.5; 55] years) and middle-aged (median age 64 [62; 67] years) men with arterial hypertension and with stable angina I-III functional classes with and without EVA. The criteria for the inclusion of patients in the subgroup with EVA were a young age of arterial hypertension debut (before 45 years), a young age of CAD debut (before 45 years), and increased vascular wall stiffness according to the cardio-ankle vascular index (according to sonography). The authors showed that in men with arterial hypertension, CAD, and EVA, the relative LTL was statistically significantly shorter than in men with arterial hypertension and CAD but without EVA (*p*-value = 0.026) [65].

Thus, in recent years, researchers and clinicians have been very interested in studying the relationship between LTL and stable CAD. The number of ongoing studies is increasing. However, the results obtained vary over a wide range, which may be due to differences in patient age, ethnicity and race, and region of residence. Nevertheless, there is no doubt that LTL is reduced in middle-aged and elderly adults developing stable CAD compared with healthy adult controls without CVD including CAD [68].

### 3.2. Leukocyte Telomere Length in Patients with Acute Myocardial Infarction

The number of LTL studies in patients with AMI is still significantly lower compared to LTL studies in patients with stable CAD. We found and analyzed five studies (Table 3). In adults with AMI, the relative LTL ranged from 0.115 CU [60] to 0.86 CU [57]; the median relative LTL was 0.62 [0.20; 0.84] CU. Differences in mean LTL in patients with AMI compared with healthy controls were not statistically significant (*p*-value = 0.089).

Russo et al. [69] found no statistically significant association between relative LTL and the risk of AMI in an Italian cohort of younger patients (≤ 48 years).

However, Gupta et al. [60] demonstrated that the relative LTL adjusted for sex, age, and body mass index was statistically significantly greater in the control group (0.792 CU) compared with AMI patients (0.115 CU, *p* -value < 0.001).

Chan et al. [71] investigated LTL in 135 patients with ACS without ST elevations who underwent percutaneous coronary intervention. The mean age of the patients was 81 ± 4 years and 64% of them were men. The mean LTL was found to be 0.47 ± 0.25 CU. Then, patients were divided into 3 groups according to the relative LTL to assess the risk of adverse clinical outcomes (death, recurrent AMI, unplanned revascularization, stroke, significant bleeding) recorded 1 year from the time of the ASC diagnosis. Long LTL was taken as 0.74 ± 0.27 CU, medium LTL as 0.42 ± 0.05 CU, and short LTL as 0.25 ± 0.27 CU. The authors found no statistically significant association between relative LTL and adverse ACS outcomes in older Chinese people.

Dlouha et al. [57] found that the mean relative LTL in 505 elderly (mean age 61 ± 9.7 years) Czech women with AMI was statistically significantly lower than those in the control group (0.86 ± 0.32 CU vs. 0.93 ± 0.38 CU; *p*-value < 0.001). However, after adjusting for age, smoking status, and type 2 diabetes mellitus, the differences between the groups were no longer significant (*p*-value = 0.25). Thus, the authors concluded to the contrary that AMI was not associated with relative LTL in Czech women.

A study by Margaritis et al. [70] determined relative LTL in AMI patients in the United Kingdom. The results were presented as median and percentiles. Short LTL was considered to be less than 0.96 CU and long LTL ≥ 0.96 CU. The authors showed that LTL is a molecular biomarker of cardiovascular outcomes after AMI regardless of patients’ ages. Also, they demonstrated that short LTL (T/S < 0.916 CU) in patients with AMI is a predictor of the high risk of all-cause mortality (*p*-value = 0.008) and mortality from CVDs within the first year after AMI (*p*-value = 0.005).

Thus, in patients with AMI, the relative LTL ranged from 0.115 CU [60] to 0.86 CU [57].

## 4. Discussion

We found and analyzed a total of 17 publications including 5 studies conducted in Russia. However, three [72,73,74] of the five Russian studies were excluded from the subsequent systematic analysis due to an alternative methodological approach to LTL measurement. The results of the 14 publications were systematized and ranked into 3 groups (adults without CVDs, adults with stable CAD, and adults with AMI), as demonstrated in Table 1, Table 2 and Table 3.

We demonstrated that in studies of the mean relative LTL in young, middle-aged, and elderly adults, the value of this molecular biomarker decreases not only in relation to the physiological biological aging of the human body but also in relation to the premature development of stable CAD [64] and the early development of AMI [60]. At the same time, the most convincing results were obtained in studies of relative LTL in middle-aged and elderly patients with stable CAD [55,58,59,61,63,64,65]. It seems important from a practical point of view because patients with stable CAD and with shortened relative LTL can have unfavorable prognoses concerning general mortality [75]. However, the prognosis of cardiovascular and cerebrovascular mortality in patients with stable CAD and with shortened LTL need to be clarified in the future.

Interestingly, the relative LTL in patients with AMI (Table 3) is shorter compared with the relative LTL in patients with CAD (Table 2) and with healthy controls (Table 1). However, we must admit that the small number of studies of relative LTL in patients with AMI does not allow us to draw any definitive conclusions.

Studies of relative LTL in patients with AMI (predominantly) and with stable CAD (to a lesser extent) are in their infancy. Such studies are still in the minority, the sample sizes are small, and the results obtained in some studies are contradictory [60,69,70,71]. The ethnic and racial heterogeneity of the samples in the publications we analyzed draws attention, which does not allow us to assess the additional influences of ethnicity and region of residence of adults with and without the studied CVDs on the absolute and relative LTL.

**Figure 3:**
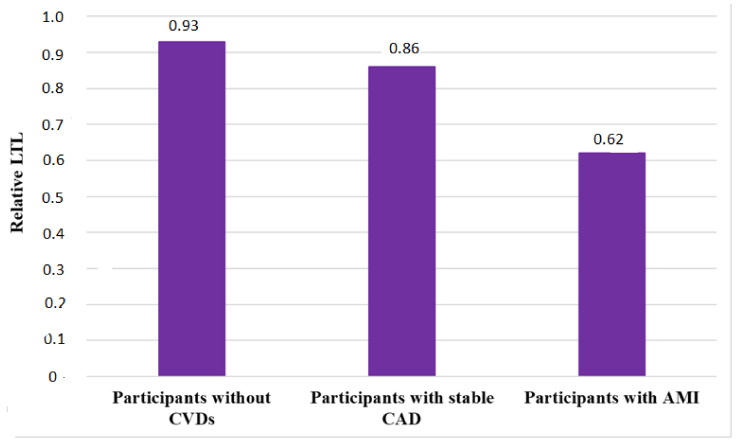
Median Relative Leukocyte Telomere Length in Patients with Coronary Heart Disease and Acute Myocardial Infarction Compared to Healthy Controls: AMI—acute myocardial infarction; CVDs—cardiovascular disease; CAD—coronary artery disease; LTL—leukocyte telomere length (CU—conventional units).

However, our systematic review of available publications demonstrated a trend toward shorter relative LTL in patients with AMI (0.62 [0.20; 0.84] CU) compared with patients with stable CAD (0.86 [0.82; 1.07] CU) and a healthy control group (0.93 [0.70; 1.10] CU) (Figure 3). However, these results did not reach statistical significance (*p*-value > 0.05). Nevertheless, this trend could be clarified by new, large, and multicenter studies with a similar design in the future.

Thus, CAD and arterial hypertension [76,77,78,79,80,81], CAD and atrial fibrillation [82,83,84,85,86,87,88,89,90,91] and CAD and vascular cognitive disorders [92,93,94,95] are common comorbid conditions with overlap syndrome. Therefore, the study of LTL seems important not only in isolated CAD and AMI but also in these syndromes of mutual aggravation.

## 5. Limitations

The limitations of this systematic review include the analysis of publications in English and Russian only. It is possible that we missed some studies published in other languages that were not represented in the databases we analyzed.

Another limitation of the review of published and available studies of relative LTL in adults with CVDs is the different methodological approaches of the investigators with regard to the study design, inclusion/exclusion criteria (e.g., age and sex of patients, ethnic group, etc.), and study duration (most studies were cross-sectional, not longitudinal). In addition, some authors used % rather than CU to estimate relative LTL.

Due to methodological problems (differences in how LTL is calculated), three Russian-language publications were excluded from the review. Thus, an interesting study is by Maximov et al. [72], in which the association of LTL with various risk factors of age-associated diseases in the Russian population was studied. The authors identified a group of patients with stable CAD in whom LTL was determined, but no recalculation of mean age and percentage by gender was performed for this group of patients, which did not allow us to include this study in our systematic review. Strajesko et al. [73] studied the relationship between risk factors for CVDs and LTL, but the authors used an alternative approach to LTL measurement, unlike the methods in the English-language publications we analyzed. Thus, the authors took 9.75 units as a short LTL and more than or equal to 9.75 units as a long LTL. Thus, the approach to determining the relative LTL in the English-language and some Russian-language publications differed significantly. In addition, the authors used three models based on multivariate linear regression analysis [73] to assess the relationship between LTL and CVD risk factors, which is of undoubted scientific interest. However, this method of statistical analysis was not used in other publications we analyzed. The authors showed that relative LTL was associated with the mean and old age of the individuals included in the study, but this publication lacked baseline data on LTL in patients with stable CAD and healthy adults. This limitation prevented us from including the authors’ findings in our systematic review. Doroshchuk et al. [74] used LTL in patients with stable CAD compared to a group of age-matched healthy volunteers as the study index. The authors demonstrated that LTL statistically significantly decreased with increasing cardiovascular mortality risk according to the Systematic Coronary Risk Evaluation (SCORE) scale, *p*-value < 0.005. However, the authors used an alternative method of measuring LTL—not in CU, but as a percentage compared to healthy controls. Due to the alternative LTL calculation methodology, the results of this study were also not included in our systematic review.

The other main concern is that relative measured LTL cannot be compared between studies, but relative measured LTLs are comparable between groups inside one study. This is due to the fact that relative LTL measured as the T/S ratio measures the relative amount of telomeric DNA (T) to a single copy gene (S), calibrated to a plate reference genomic DNA sample. Therefore, and as this reference sample is unique for each study, the results on relative quantified LTLs are not directly comparable between studies.

The association between LTL and EVA has had a limited focus in the present review and we therefore plan to include it in a future review of the relationship between EVA and LTL in young, middle-aged, and elderly patients.

## 6. Conclusions

Despite the scientific and clinical significance of the analyzed studies on relative LTL as a molecular biomarker of CVDs, their translation into real clinical practice is difficult due to disparities in the design and methodology of the analyzed studies, including the studies on cell cultures and humans, as well as the differences in samples by gender, age, race, and ethnicity. The authors believe that clinical studies of the role of relative LTL in adult patients with CAD are the most promising and require large multicenter studies with a unified design and methodology.

## Figures and Tables

**Table 1:** Mean Relative Telomere Length of Leukocytes in Adults without Cardiovascular Disease.

Authors [References]	Study Characteristics	Group Characteristics	Participants (n)	Age, in Years (M ± SE or Me [P25; P75])	Sex (Male/Female, %)	Method	Telomere Length	
							Absolute, bp	Relative (T/S Ratio), CU
Williet et al., 2010 [55]	Prospective, population-based study	Austrians without CAD	712	61.8 ± 10.8	47.6/52.4	Real- time quantitative PCR	N/A	1.52 ± 0.81
Dlouha et al., 2016 [57]	Observational, cross-sectional case-control study	Czechs without CAD	642	50 ± 2.7	0/100	Real-time quantitative PCR	N/A	0.93 ± 0.38
Tian et al., 2017 [58]	Observational, cross-sectional study	Chinese without CAD	128	48.5 ± 7.33	57.8/42.2	Real- time quantitative PCR	N/A	1.1 ± 0.57
Pejenaute et al., 2020 [59]	Observational, cross-sectional study	Spaniards without CAD	389	54 ± 1	80/20	Real- time quantitative PCR	8591 ± 84	N/A
Gupta et al., 2020 [60]	Observational, cross-sectional study	Indians without CVDs	77	34.38 ± 5.86	75/25	Real- time quantitative PCR	N/A	0.792
Starnino et al., 2021 [61]	Observational, cross-sectional study	Canadians without CVDs	25	55.68 ± 0.19	56/44	Real- time quantitative PCR	N/A	0.94 ± 0.15
Mazidi et al., 2021 * [62]	Mendelian randomized trial	British without CAD	20	22.3 ± 1.8	100/0	Real-time quantitative PCR	12 420 ± 80	N/A
Mazidi et al., 2021 * [62]	Mendelian randomized trial	British without CAD	20	62.75 ± 2.1	100/0	Real-time quantitative PCR	6 380 ± 60	N/A
Hassler et al., 2021 ** [56]	Observational, cross-sectional study	Austrians without CVDs	90	40.77 ± 11.62	100/0	Real- time quantitative PCR	N/A	0.7 ± 0.28
Hassler et al., 2021 ** [56]	Observational, cross-sectional study	Austrians without CVDs	90	44.71 ± 10.96	0/100	Real- time quantitative PCR	N/A	0.69 ± 0.31

**Notes:** bp—base pairs; CU—conventional units; N/A—no data; PCR—polymerase chain reaction. * There were two groups in the Mazidi study. One group of patients is young (mean age 22 years old), and the other group is elderly (mean age 62.75 years old). For each of the groups, this author calculated the relative LTL. ** There were two groups in the Hassler study: men and women without CVDs. Separately for each group, the author measured the relative LTL. Therefore, these results are listed in two rows of Table 1.

**Table 2:** Mean Relative Telomere Length of Leukocytes in Adults with Stable Coronary Artery Disease.

Authors [References]	Study Characteristics	Group Characteristics	Participants (n)	Age, in Years (M ± SE or Me [P25; P75])	Sex (Male/Female, %)	Method	Telomere Length	
							Absolute, bp	Relative (T/S Ratio), CU
Williet et al., 2010 [55]	Prospective, population-based study	Austrians with a stable CAD	88	70 ± 10.5	63.6/26.4	Real- time quantitative PCR	N/A	1.13 ± 0.52
Yakhontov et al., 2017 * [64]	Observational, cross-sectional study	Russians with stable CAD I-III FC	59	52 [46.5; 55]	100/0	Real- time quantitative PCR	N/A	0.84 [0.2; 1.9]
Yakhontov et al., 2017 * [64]	Observational, cross-sectional study	Russians with stable CAD I-III FC	47	64 [62; 67]	100/0	Real- time quantitative PCR	N/A	0.3 [0.09; 1.2]
Hammadah et al., 2017 [63]	Observational, cross-sectional study	Canadians with stable CAD	566	63 ± 9,0	63.6/26.4	Real- time quantitative PCR	N/A	0.82 ± 0.14
Tian et al., 2017 [58]	Observational, cross-sectional study	Chinese with premature CAD	128	48.6 ± 7.26	57.8/42.2	Real- time quantitative PCR	N/A	0.88 ± 0.86
Yakhontov et al., 2018 [65]	Observational, cross-sectional study	Russians with essential hypertension and stable CAD I-III FC	43	52 [46.5; 55.0]	100/0	Real- time quantitative PCR	N/A	0.7 [0.12; 0.92]
Pejenaute et al., 2020 [59]	Observational, cross-sectional study	Spaniards with coronary atherosclerosis	116	61 ± 1	88/12	Real- time quantitative PCR	8315 ± 98	N/A
Starnino et al., 2021 [61]	Observational, cross-sectional study	Canadians with stable CAD	598	66.13 ± 6.25	80.6/19.4	Real- time quantitative PCR	N/A	0.83 ± 0.18

**Notes:** bp—base pairs; CAD—coronary artery disease; CU—conventional units; N/A—no data; PCR—polymerase chain reaction; FC—functional class. * There were two groups in Yakhontov’s study: middle-aged patients with stable CAD (mean age—52 years) and elderly patients with stable CAD (mean age—64 years). LTL was determined for each group. Therefore, both groups studied by Yakhontov are included in Table 2.

**Table 3:** Mean Relative Telomere Length of Leukocytes in Adults with Acute Myocardial Infarction.

Authors [References]	Study Characteristics	Group Characteristics	Participants (n)	Age, in Years (M ± SE or Me [P25; P75])	Sex (Male/Female, %)	Method	Telomere Length	
							Absolute, bp	Relative (T/S Ratio), CU
Russo A. et al., 2012 [69]	Observational, open, cross-sectional, longitudinal study.	Italians with AMI	199	40.1 ± 5	89.4/10.6	Real- time quantitative PCR	N/A	0.77 ± 0.2
Dlouha, D. et al., 2016 [57]	Observational, cross-sectional case-control study	Czechs with AMI	505	61 ± 9.7	0/100	Real- time quantitative PCR	N/A	0.86 ± 0.32
Margaritis, M. et al., 2017 [70]	Observational, open, cross-sectional, longitudinal study	British with AMI	290	63 ± 12.7	85.2/14.8	Real- time quantitative PCR	N/A	1.08 [0.41—2.66] *
Gupta M.D. et al., 2020 [60]	Observatio-nal, open, cross-sectional study	Indians with AMI	77	35.33 ± 6.22	84.4/15.6	Real- time quantitative PCR	N/A	0.115
Chan D. et al., 2020 [71]	Prospective, observation, cohort, longitudinal study.	British with AMI	135	81 ± 4	64/36	Real- time quantitative PCR	N/A	0.47 ± 0.25

**Notes:** bp—base pairs; CU—conventional units; N/A—no data; PCR—polymerase chain reaction; *—median [P10—P90].

## Data Availability

Not applicable.
